# Postprandial Plasma and Whole Blood Amino Acids Are Largely Indicative of Dietary Amino Acids in Adult Dogs Consuming Diets with Increasing Whole Pulse Ingredient Inclusion

**DOI:** 10.1016/j.tjnut.2024.07.023

**Published:** 2024-07-16

**Authors:** Sydney Banton, Pawanpreet Singh, Dave J Seymour, Jennifer Saunders-Blades, Anna K Shoveller

**Affiliations:** 1 Department of Animal Biosciences, University of Guelph, Guelph, Ontario, Canada; 2 Trouw Nutrition R&D, Amersfoort, Netherlands; 3 Champion Petfoods Holding, Morinville, Alberta, Canada

**Keywords:** canine, pulse ingredients, grain-free diets, foodomics, amino acids, partial least squares regression, phospholipids

## Abstract

**Background:**

Pulse ingredients often replace grains in grain-free dog diets owing to their high-protein content. However, research to ascertain the benefit of this modification is limited.

**Objectives:**

This study aimed to correlate food compounds in 1 corn-inclusive control diet and 3 grain-free diets with increasing inclusions of whole pulses (≤45%; Pulse15, Pulse30, and Pulse45), formulated to meet similar macronutrient and micronutrient targets with postprandial amino acids (AAs) in healthy dogs >20 wk.

**Methods:**

Diets were analyzed for biochemical compounds using tandem mass spectrometry. Twenty-eight outdoor-housed, healthy, adult Siberian Huskies were allocated to diet, and meal responses were analyzed at baseline and weeks 2, 4, 8, 16, and 20 with samples collected at fasted and 15, 30, 60, 90, 120, and 180 min after meal presentation. Blood AAs were analyzed by ultra performance liquid chromatography and differences across week, treatment, and time postmeal were analyzed in SAS Studio. Partial least squares regression was performed in SAS Studio using biochemical compounds in the diet as predictor variables and blood AAs as response variables.

**Results:**

In plasma, Pulse45 had ∼32% greater postprandial Asn than Pulse15, and the control diet had ∼34% greater postprandial Leu and ∼35% greater Pro than Pulse15 (*P* < 0.05). In whole blood, Pulse30 had ∼23% greater postprandial Lys than the control diet, and the control diet had ∼21% greater postprandial Met and ∼18% greater Pro than Pulse45 and Pulse30, respectively (*P* < 0.05). Several phospholipids were correlated with postprandial AAs. Compounds in the urea cycle and glycine and serine metabolism were more enriched (*P* < 0.05) in plasma and whole blood, respectively.

**Conclusions:**

In macronutrient-balanced and micronutrient-balanced canine diets that differ in their inclusion of corn-derived compared with pulse-derived ingredients, postprandial changes in circulating AAs are largely indicative of the dietary AAs. This helps further our understanding of AA metabolism in healthy dogs fed grain-free diets.

## Introduction

Pulses are the dried seeds harvested from legumes such as peas, chickpeas, beans, and lentils. As the global population increases and with it the demand for protein, a variety of dietary protein sources are needed. Pulses contain ∼17% to 30% dietary protein [1], making them an excellent plant-based protein alternative. They are typically high in the amino acids (AAs) Lys but low in Met, Cys and often Trp [2] when compared with the current recommended allowances for these AAs for dogs [3]. In addition, pulses have several other features that make them a desirable alternative protein. For example, the starches in pulses typically have a higher content of amylose than cereals, which helps make their digestion slower, giving them a lower glycemic index [4]. This, combined with their high-dietary fiber and low-fat content, make them especially useful in managing satiety and weight in humans [4].

Pulses have been used in the pet food industry for years, previously in combination with cereal grains, as the 2 ingredients have complimentary AA profiles [3]. However, there has recently been an increase in the prevalence of grain-free diets. This has led to the removal of the inclusion of cereal grains and the increase in the inclusion pulse ingredients (>20%) without supporting data [5]. Although there is an abundance of digestibility data for pulse ingredients in other species [[6], [7], [8]], little consideration has been given to whether or not these digestibility values support canine AA requirements. Using the cecectomized rooster as a model for dogs [9], Reilly et al. [10] reported indispensable AA digestibility scores for yellow peas, green lentils, and chickpeas, between 80% and 95% [on a dry matter basis (DMB)] for all AAs, except Met. The low-Met content and digestibility/bioavailability in dogs fed diets containing pulses was initially suggested to be a potential cause of Tau deficiency that may lead to the development of nutritionally mediated dilated cardiomyopathy (DCM) in dogs [5]. This has led to dog owners and veterinarians remaining skeptical in feeding these diets given this proposed link to DCM. However, multiple studies have shown that healthy dogs consuming grain-free, pulse-inclusive diets do not develop a Tau deficiency, as assessed by plasma and whole blood Tau concentrations [[11], [12], [13], [14]]. AA metabolism is dynamic, and in order to understand the broader implications of how pulse ingredients affect canine metabolism, AAs in both the diet and blood of dogs consuming the diet need to be investigated over time as well as after a meal. Although it is difficult to assess AA transport and interactions with splanchnic tissues in companion animals owing to cost and ethical constraints, postprandial AAs in the blood gives us a better idea of AA availability than digestibility alone.

A novel approach to understanding metabolism on a more integrated level is metabolomics, which measures hundreds of small molecules in a biological sample [15]. This has been applied to companion animal research because it provides data for hundreds of molecules using a small sample volume [[16], [17], [18], [19]]. The same approach can be applied to food, known as foodomics [20]. To our knowledge, only 1 study has applied foodomics to grain-free, pulse-inclusive diets. Smith et al. [21] reported 24 AA-related compounds, 20 plant compounds, and 18 lipid compounds were higher and 8 vitamins were lower in commercial grain-free, pulse-inclusive canine diets than those in grain-inclusive canine diets. The authors concluded that some of these compounds may be used as biomarkers and may contribute to the development of DCM in dogs [21]. However, 9 commercial diets were selected to represent these 2 groups with no control for macronutrients or micronutrients among diets. Therefore, comparing food compounds between diets of varying protein, fat, and fiber contents becomes difficult and causal conclusions cannot be made. In order to understand the chemical differences between grain-free and grain-inclusive diets, careful consideration needs to be made, including formulating research diets that are balanced for macronutrients and micronutrients and using the same ingredients that are processed under similar conditions. Only controlled studies can pinpoint significant differences among diets containing different ingredients.

The primary objective of the present study was to quantify the plasma and whole blood meal response in healthy adult dogs consuming either a corn-inclusive control diet or grain-free diets with increasing inclusions of whole pulse ingredients (≤45%; Pulse15, Pulse30, and Pulse45) that were formulated to meet similar macronutrient and micronutrient targets and fed for 20 wk. The secondary objective was to correlate food compounds (measured via foodomic analysis) in the diets with fasted and postprandial plasma and whole blood AA concentrations in the dogs. Based on the AA profile of each diet (measured via nutritional analysis), we hypothesized that dogs fed the control diet would have higher postprandial plasma and whole blood Met and Leu concentrations than dogs fed the pulse diets. In addition, dogs fed the pulse diets would have higher postprandial plasma and whole blood Arg and Lys concentrations than dogs fed control diet as a result of the nutrients in the diet they are consuming. In addition, we hypothesized that the AA-related food compounds in the diet that are involved in Met, Leu, Arg, and Lys metabolism would be associated with plasma and whole blood AA concentrations in the dogs. This study was conducted as part of the study by Singh et al. [14] where the effect of feeding these diets on cardiac health was evaluated.

## Methods

### Animals and feeding

All experimental procedures were approved by the University of Guelph’s Animal Care Committee (AUP #4553). Twenty-eight privately owned Siberian huskies (15 males, 9 neutered; 13 females, 9 spayed) were housed outdoors at Rajenn Siberian Huskies in free-run kennels (3.5–80 m^2^) with ≤8 dogs per kennel. Dogs were between the ages of 1 and 10 y, with a mean (±SD) age of 5.3 ± 2.8 y and had a mean (±SD) body weight of 23.3 ± 3.7 kg.

Before the start of the study, dogs were acclimated to being fed once daily at 16:00 h. Dogs were fed an adaptation diet (Acana Healthy Grains Dog Food Red Meat; Champion Petfoods Holding) for 4 wk (week −4 to −1) according to historical feeding records for body weight maintenance. Dogs were individually tethered during feeding. They were given 1 h to eat, and all remaining food was weighed and recorded daily. All dogs had ad libitum access to fresh water. Throughout the study, dogs were weighed weekly, and feed intake was adjusted to maintain body weight at week 0.

### Diets and study design

Four experimental extruded diets were formulated and produced by Champion Petfoods Holding to meet similar macronutrient and micronutrient targets and exceed the nutrient recommendations set out by the Association of American Feed Control Officials (AAFCO) [22] for adult dogs at maintenance. All diets were made using a single screw extruder with similar barrel temperature ranges and dried under similar temperatures, ranging from 110 to 135°C. The diets were analyzed by Silliker Canada, an ISO 17025 accredited laboratory (Mérieux NutriSciences). The control grain-inclusive diet contained 33% corn, 12% corn gluten meal, and 0% whole pulse ingredients, and the grain-free, whole pulse-containing diets were formulated with 0% corn and 5%, 10%, or 15% of each of the following: green and yellow pea flour, pinto bean flour, and chickpea and lentil flour, respectively (Table 1). Chicken meal was used as the main animal protein source in each diet and decreased as pulse inclusion increased to achieve ∼32% crude protein on an as-fed basis. Similarly, pea starch decreased as pulse inclusion increased. The diets were formulated to achieve a similar crude protein and upper limit of pulse inclusion that are commonly seen in the marketplace. All other ingredients, including vitamins and minerals, were kept the same across diets. All diets were processed and manufactured with the same ingredients.

**TABLE 1:** Ingredient composition, proximate analysis, and nutrient composition of control grain-inclusive diet and 3 grain-free diets with increasing pulse inclusion (Pulse15, Pulse30, and Pulse45) analyzed via AOAC standard protocols, presented on an as-fed basis^1^.

	Control	Pulse15	Pulse30	Pulse45
Ingredient				
Whole grain corn	33.00	—	—	—
Corn gluten meal	12.00	—	—	—
Chicken meal	25.00	33.00	27.25	25.00
Pea starch	2.20	24.20	14.94	2.20
Whole green and yellow peas flour	—	5.00	10.00	15.00
Whole pinto beans flour	—	5.00	10.00	15.00
Whole chickpeas and lentils (50:50) flour	—	5.00	10.00	15.00
Fresh, mechanically separated chicken	10.00	10.00	10.00	10.00
Chicken fat	7.50	7.50	7.50	7.50
Ground *Miscanthus* grass	2.00	2.00	2.00	2.00
Natural chicken flavor (dry)	1.50	1.50	1.50	1.50
Natural chicken flavor (liquid)	2.50	2.50	2.50	2.50
Salt	2.50	2.50	2.50	2.50
Potassium chloride	0.75	0.75	0.75	0.75
Kelp	0.25	0.25	0.25	0.25
Proximate analysis (%)				
Moisture	9.58	10.60	11.20	10.20
Crude protein	31.92	32.72	31.52	32.42
Crude fat	15.46	13.50	13.59	14.28
Total dietary fiber	5.50	6.65	8.21	8.31
Nitrogen-free extract (NFE, calculated)^2^	33.83	31.50	31.71	30.83
Ash	7.09	8.62	8.70	8.84
Calculated metabolizable energy (kcal/kg)^3^	3615.31	3395.32	3368.05	3427.26
AA (%)				
Arginine	1.84	2.27	2.21	2.31
Lysine	1.43	1.98	1.94	1.98
Methionine	0.76	0.63	0.59	0.58
Phenylalanine	1.73	1.53	1.55	1.65
Cysteine	0.48	0.46	0.45	0.45
Histidine	0.89	0.91	0.89	0.91
Isoleucine	1.19	1.28	1.24	1.28
Leucine	3.21	2.57	2.33	2.43
Threonine	1.18	1.30	1.25	1.30
Tryptophan	0.21	0.30	0.31	0.29
Tyrosine	0.98	0.85	0.82	0.85
Valine	1.39	1.50	1.45	1.51
Taurine^4^	0.08	0.13	0.12	0.11
Fiber				
Total insoluble fiber (%)	4.75	5.78	6.19	6.36
Total soluble fiber (%)	0.75	0.87	2.02	1.95
Oligosaccharides (mg/g)				
Sucrose	4.90	10.87	12.42	12.09
Raffinose	0.46	3.12	3.91	2.49
Stachyose	0.81	8.31	7.96	10.3
Verbascose	0.33	4.63	3.51	2.96
Total starch (%)	22.03	16.95	16.77	15.89

1 All diets were formulated with equal inclusion of vitamin and mineral premixes: vitamin B premix canine (0.2%); vitamin A, D, and E (0.2%); choline chloride (1.5%); zinc (0.1%); vitamin B-5 (0.05%); selenium (0.05%); natural antioxidant liquid (0.04%); natural antioxidant dry (0.02%); and copper (0.01%).

2 NFE = 100 − (moisture + protein + fat +fiber + ash).

3 Metabolizable energy = [8.5 kcal metabolizable energy (ME) × g crude fat] + (3.5 kcal ME × g crude protein) + (3.5 kcal ME × g nitrogen-free extract) × 10.

4 Taurine was analyzed separately, 7 mo later.

Dogs were switched on to 1 of the 4 diets on the first day of week 0 after the 4-wk adaptation period. Dogs were assigned to 1 of the 4 groups (*n* = 7 per group) balanced for echocardiographic ejection fraction [14], age, sex, neuter status, and body weight. One of the 4 diets was then randomly assigned to each group, and the dogs were fed the diets for 20 wk.

### Foodomics

Diets were analyzed at the Metabolomics Innovation Centre for >200 biochemical compounds. AAs, acylcarnitines, uremic toxins, biogenic amines and derivatives, glycerophospholipids, sphingolipids, and sugars were analyzed using the Metabolomics Innovation Centre Prime Food Assay. Briefly, 100 mg each diet was added to 1 mL of 3:1 hexane:MeOH and then vortexed, shaken, and sonicated on ice. Then, another 650 μL of 3:1 H_2_O:MeOH was added, vortexed, and centrifuged at 10,000 × *g* at 4°C for 10 min. The top layer was removed, and nitrogen purge was used to dry the removed sample for 20 min. Samples were then reconstituted with acetonitrile. Samples were analyzed with a custom assay using a ABSciex 4000 Qtrap tandem mass spectrometry instrument (Applied Biosystems/MDS Analytical Technologies) equipped with an Agilent 1260 series ultra–high-performance liquid chromatography (UHPLC) system (Agilent Technologies). For polyphenol analysis, 7.5 mg each diet was added to 10 μL of a mixture of 10 stable isotope-labeled internal standards and 250 μL of 80% methanol. Samples were vortexed and centrifuged at 14,000 × *g* for 5 min. Supernatant was filtered and made ready for analysis using a Thermo Scientific Vanquish UHPLC system and an Agilent reversed-phase Zorbax Eclipse XDB C18 column (3.0 mm × 100 mm, 3.5-μm particle size, 80 Å pore size) with an ACQUITY UPLC CSH C18 VanGuard Precolumn (2.1 mm × 5 mm, 1.7-μm particle size, 130 Å pore size). Finally, for the water-soluble vitamin analysis, each diet was dissolved in water at a concentration of 10 mg/mL, vortexed, and then centrifuged at 18,000 × *g* at 4°C for 15 min. Supernatant was extracted and diluted to 1 mg/mL, and the stable isotope-labeled internal standards mixture was added along with 60 μL trichloroacetic acid. Samples were vortexed, left on ice for 1 h, and then centrifuged at 13,000 × *g* for 20 min. Samples were analyzed using an Agilent 1260 series UHPLC system (Agilent Technologies) coupled with an AB Sciex QTRAP 4000 mass spectrometer (Sciex Canada) with an Agilent reversed-phase Zorbax Eclipse XDB C18 column (3.0 mm × 100 mm, 3.5-μm particle size, 80 Å pore size) coupled to a Phenomenex SecurityGuard C18 precolumn (4.0 mm × 3.0 mm).

### Meal response

On weeks 2, 4, 8, 16, and 20, a 3-h meal response was performed with 13 dogs 1 day and then 14 dogs the next. Each dogs’ forearm was shaved and topical anesthetic (eutectic mixture of local anesthetics (EMLA) cream; 2.5% lidocaine and 2.5% prilocaine; Astra Pharmaceuticals) was applied. After 20 min, the leg was cleaned with 70% alcohol and then 4% chlorhexidine, and 20-gauge cephalic catheters (Insyte-W 20 G × 1.1; Becton Dickinson Canada) were placed. After being deprived of food for ∼22 h, a 5-mL fasted sample (time 0) was taken immediately after placement. A 3-way stopcock (Cardinal Health Canada) was attached to each catheter and flushed with 0.5 mL of 50 United States Pharmacopeia (USP)/mL heparinized saline and locked with 0.5 mL of 100 USP/mL heparinized saline (Sandoz Canada). If a catheter could not be placed, a fasted sample and 3 postmeal samples (30, 60, and 90 min) were taken via cephalic venipuncture. Once catheters were placed, dogs were fed 75% of their daily intake, 10 min apart from one another starting at ∼14:00 h. If dogs did not consume their food, they were removed from the meal response, and only a fasted sample was used for analysis. Immediately after the dog started eating, the timer was started, and samples were collected at 15, 30, 60, 90, 120, and 180 min after starting to eat the meal. For every sample, 3–5 mL of blood was taken, placed in a 10-mL sodium heparin tube (Becton Dickinson Canada), and placed on ice. After every sample was taken, the catheter was flushed with 0.5 mL of 50 USP/mL heparinized saline and locked with 0.5 mL of 100 USP/mL heparinized saline via the same port on the 3-way stopcock; therefore, no blood was discarded. At each time point, a 0.5-mL aliquot of whole blood was removed and stored on ice. The remaining blood was centrifuged at 4 °C at 12,000 × g for 15 min. Plasma was separated and stored on ice. At the end of each sampling day, all samples were moved to a −80 °C freezer until analysis.

### Plasma and whole blood AA analysis

Plasma and whole blood free AA concentrations were analyzed using UPLC (Waters Corporation) using the method, adapting from the study by Bidlingmeyer et al. [23], was described in the study by Banton et al. [24]. Before analysis, whole blood was frozen at −80°C and thawed twice to lyse open red blood cells.

Total plasma homocysteine and glutathione (GSH) were analyzed using UPLC using the method as previously described in the study by Banton et al. [24], which was modified from Vester and Rasmussen [25] and Pfeiffer et al. [26].

### Statistical analysis

Plasma and whole blood AA data were analyzed as doubly repeated measures using the MIXED procedure of SAS Studio (v 9.4; SAS Institute Inc.) where week and time postmeal were repeated with dog as the subject. In the statistical model, the effect of treatment, time postmeal, week, treatment × time postmeal interaction, and treatment × week interaction were evaluated. The 3-way interaction among treatment, time postmeal, and week was not evaluated owing to lack of biological relevance related to the objectives of the study. For each AA, model assumptions were assessed through residual analysis, and if assumptions were violated, a modification to the covariance structure was made or log transformation was performed. Three covariance structures that are appropriate for multivariate repeated measures were investigated {direct product unstructured [UN@UN], direct product first-order autoregressive [UN@AR(1)], and direct product compound symmetry [UN@CS]} and the one with the lowest Akaike information criterion (AIC) value was used. Residuals for plasma serine were not normally distributed and thus were log transformed before analysis. In addition, preplanned orthogonal contrasts were performed to assess the strength of linear or quadratic relationships across treatments, specifically to address increasing whole pulse inclusion. Initial analysis was performed using week −1 as a covariate; however, owing to missing data from several dogs in week −1, it was removed as a covariate if there were no differences among treatments at week −1. This was true for all AAs except for whole blood Tau; therefore, week −1 was included as a covariate for whole blood Tau analysis. Correlations between the AAs measured via foodomic analysis and AAs measured via standard nutritional analysis, as well as both methods with plasma and whole blood AAs, were performed using the correlation procedure in SAS Studio. Means were separated using the Tukey–Kramer adjustment, and statistical significance was declared at *P* ≤ 0.05.

Partial least squares (PLS) regression was performed using the PLS procedure of SAS Studio (SAS Institute) because there were many more predictor variables than response variables. Three separate PLS regressions were performed using all plasma, all whole blood, or just the AA involved in sulfur amino acid (SAA) metabolism (plasma Met, Tau, homocysteine, cystine, Ser, glycine, and GSH) as the response variables and the >200 food compounds as predictors. The effect of treatment, time postmeal, and week were included in the statistical model. An initial PLS regression was performed and each predictor variable assigned a variable importance in projection (VIP) score. After the initial PLS regression fitting, the predictor variables with a VIP score in the bottom 25th percentile (VIP < 0.97) were removed, and a second PLS regression was performed with the remaining predictor variables. Leave-1-out crossvalidation was requested using the CV = ONE option, and any missing values imputed using the built-in expectation-maximization algorithm. In addition, metabolite set enrichment analysis (MSEA) was performed in MetaboAnalyst (version 5.0) using each list of compounds from the abovementioned PLS regression with VIP > 1.00. The Small Molecule Pathway Database (smpdb.ca) was used as the reference metabolite database. The Holm–Bonferroni correction was used to account for multiple comparisons, and statistical significance was declared at *P* ≤ 0.05.

## Results

### Plasma and whole blood AA meal response

Of all dogs across all meal challenges, only 5 consumed their meal within 60 min, the rest ate everything before the first 15-min sample. Some dogs did not consume their meal, a catheter could not be placed, or they were receiving antibiotics prescribed by the dog’s regular veterinarian because of an open wound and infected nails, unrelated to the study, and were therefore removed from the meal challenge. The following are the dogs that were removed from the meal challenge: baseline, 4 males (2 from Pulse45, 1 from Pulse30, and 1 from Pulse15) and 2 females (2 from Pulse45); week 2, 3 males (3 from Pulse45); week 4, 2 males (2 from Pulse45); week 8, 4 males (2 from Pulse45, 1 from Pulse30, and 1 from Pulse15); week 16, 2 males (1 from Pulse45 and 1 from Pulse30) and 3 females (2 from Pulse45 and 1 from the control diet); and week 20, 3 males (2 from Pulse45 and 1 from Pulse30) and 3 females (2 from Pulse15 and 1 from Pulse45).

**FIGURE 1:**
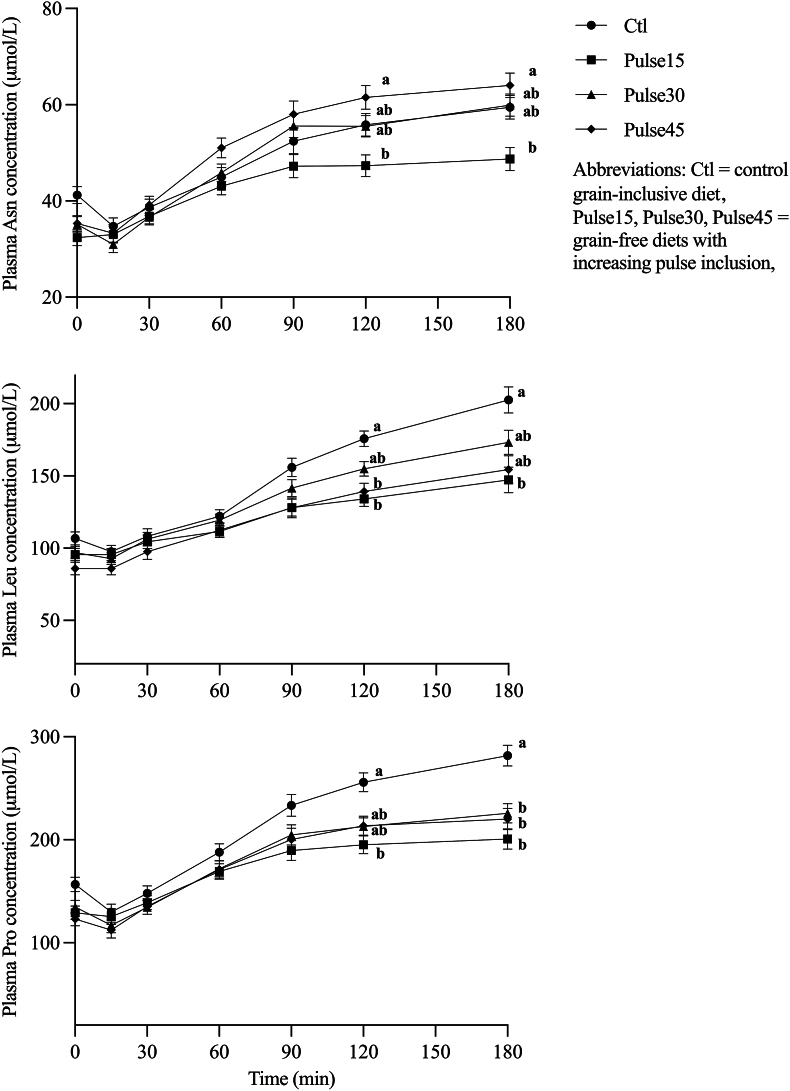
Mean plasma asparagine (top), leucine (middle), and proline (bottom) concentrations (nmol/mL) in dogs consuming control, Pulse15, Pulse30, or Pulse45, over time. Values are presented as lsmeans ± SEM. Time points that do not share a common letter are significantly different at *P* < 0.05.

**FIGURE 2:**
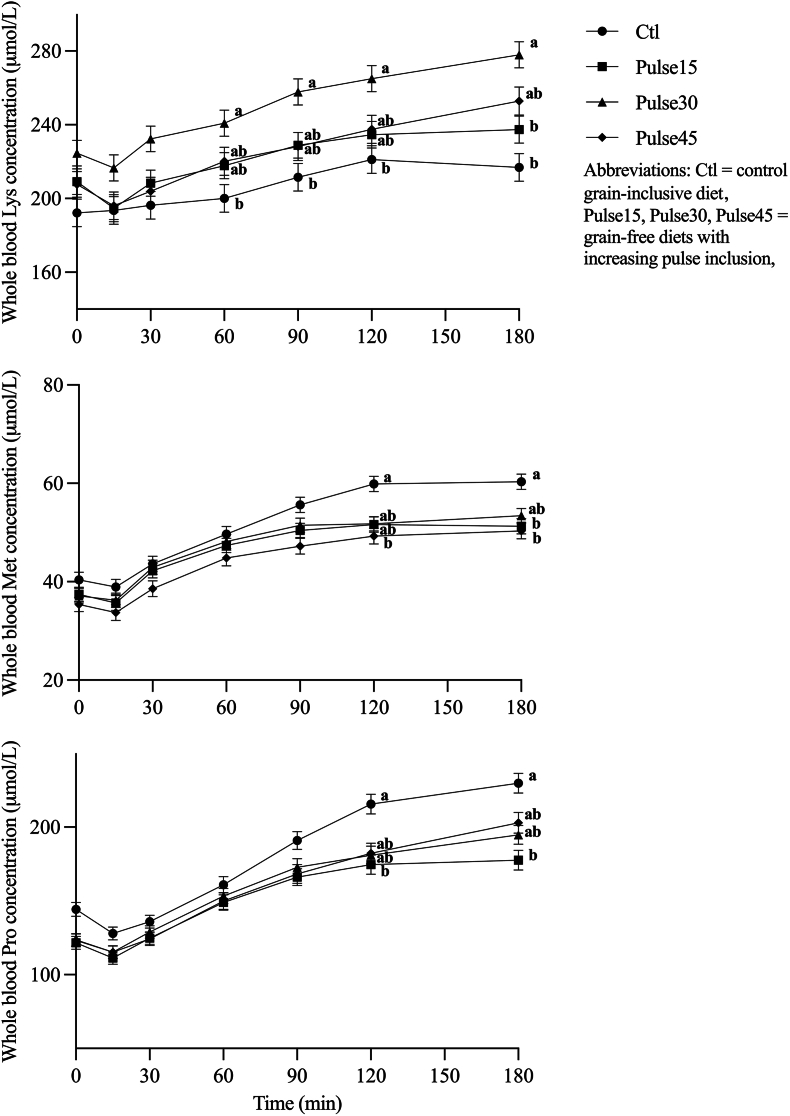
Mean whole blood lysine (top), methionine (middle), and proline (bottom) concentrations (nmol/mL) in dogs consuming control, Pulse15, Pulse30, or Pulse45, over time. Values are presented as lsmeans ± SEM. Time points that do not share a common letter are significantly different at *P* < 0.05.

Feed intake and body weight were not different among treatments or over time (*P* > 0.05), as reported in a previous study [14]. The effects of treatment, time postmeal, and week are displayed in Table 2 for all plasma AAs and Table 3 for all whole blood AAs. There was no treatment × week effect for any AA (*P* > 0.05). Plasma Asn, Leu and Pro had a significant treatment × time postmeal interaction effect (*P* < 0.05) (Figure 1). Plasma Asn was greater in Pulse45 than that in Pulse15 at 120 and 180 min postmeal (*P* < 0.05). Plasma Leu was greater in the control diet than that in Pulse15 and Pulse45 at 120 min and greater than that in Pulse15 at 180 min postmeal (*P* < 0.05). Plasma Pro was greater in the control diet than that in Pulse15 at 120 min and greater than that in all 3 pulse diets at 180 min postmeal (*P* < 0.05). Whole blood Lys, Met, and Pro also had significant treatment × time postmeal interaction effects (*P* < 0.05) (Figure 2). Whole blood Lys was greater in Pulse30 than that in the control diet at 60, 90, and 120 min and greater than that in the control diet and Pulse15 at 180 min postmeal (*P* < 0.05). Whole blood Met was greater in the control diet than that in Pulse45 at 120 min and greater than that in Pulse15 and Pulse45 at 180 min postmeal (*P* < 0.05). Whole blood Pro was greater in the control diet than that in Pulse15 at 120 and 180 min postmeal (*P* < 0.05).

**TABLE 2:** Plasma AA concentrations (lsmeans ± SEM nmol/mL) across the main effects of treatment (control, Pulse15, Pulse30, and Pulse45), time (0-fasted to 180 min), and week (2–20).

AA	Treatment				Time (min)							Week					*P*		
	Control	Pulse15	Pulse30	Pulse45	0	15	30	60	90	120	180	2	4	8	16	20	Treatment	Time	Week
*n*	197	211	222	164	129	108	111	114	114	110	108	172	167	160	150	145			
Ala	508 ± 18^a^	410 ± 17^b^	452 ± 16^a^	430 ± 18^b^	416 ± 11^d^	382 ± 11^e^	410 ± 11^d^	472 ± 11^bc^	504 ± 11^a^	497 ± 12^ab^	468 ± 12^c^	440 ± 12^ab^	442 ± 11^ab^	422 ± 11^b^	483 ± 19^a^	464 ± 18^ab^	0.001	<0.001	0.028
Arg	109 ± 7^b^	135 ± 6^a^	141 ± 6^a^	151 ± 7^a^	117 ± 4^cd^	109 ± 4^d^	121 ± 4^c^	139 ± 4^b^	152 ± 4^a^	152 ± 4^a^	149 ± 4^ab^	117 ± 4^b^	118 ± 4^b^	124 ± 4^b^	155 ± 8^a^	157 ± 6^a^	0.004	<0.001	<0.001
Asn^1^	—	—	—	—	—	—	—	—	—	—	—	42.1 ± 1^b^	43.0 ± 1^b^	41.9 ± 1^b^	49.7 ± 2^a^	51.6 ± 2^a^	—	—	<0.001
Cystine	19.2 ± 1	19.2 ± 1	18.3 ± 1	17.6 ± 1	17.1 ± 0.6^b^	18.2 ± 0.6^a^	19.3 ± 0.6^a^	18.9 ± 0.6^a^	19.1 ± 0.6^a^	18.7 ± 0.6^a^	18.7 ± 0.6^a^	19.8 ± 1^a^	19.8 ± 0.8^a^	16.1 ± 1^b^	19.3 ± 1^ab^	17.9 ± 0.8^ab^	0.650	<0.001	0.021
Gln	752 ± 20^a^	679 ± 18^bc^	674 ± 18^c^	745 ± 20^ab^	700 ± 10^abc^	690 ± 9^c^	708 ± 12^bc^	732 ± 13^a^	734 ± 13^ab^	718 ± 12^abc^	704 ± 11^c^	708 ± 18	702 ± 13	701 ± 13	741 ± 21	710 ± 13	0.004	0.004	0.461
Glu	67.8 ± 4	69.9 ± 4	73.5 ± 4	70.0 ± 4	74.4 ± 2^a^	66.3 ± 2^c^	67.7 ± 2^bc^	68.7 ± 2^bc^	70.9 ± 2^ab^	71.4 ± 2^ab^	72.7 ± 2^a^	64.7 ± 2^ab^	64.9 ± 2^ab^	61.3 ± 2^b^	81.2 ± 6^a^	79.4 ± 5^a^	0.766	<0.001	0.003
Gly	301 ± 14	292 ± 14	292 ± 13	312 ± 14	277 ± 9^c^	248 ± 8^d^	262 ± 7^cd^	305 ± 8^b^	338 ± 10^a^	334 ± 8^a^	331 ± 8^a^	261 ± 7^b^	266 ± 9^b^	276 ± 8^b^	346 ± 21^a^	346 ± 21^a^	0.727	<0.001	0.002
GSH	5.57 ± 0.4^b^	8.16 ± 0.4^a^	6.94 ± 0.4^a^	7.80 ± 0.4^a^	7.46 ± 0.2^ab^	7.50 ± 0.2^a^	7.30 ± 0.2^ab^	7.12 ± 0.2^bc^	6.91 ± 0.2^cd^	6.74 ± 0.2^d^	6.80 ± 0.2^cd^	6.32 ± 0.2^c^	6.83 ± 0.3^bc^	8.14 ± 0.4^a^	7.30 ± 0.3^ab^	6.98 ± 0.3^abc^	<0.001	<0.001	0.009
His	98.8 ± 2^ab^	95.2 ± 2^b^	105 ± 2^a^	97.0 ± 2^ab^	90.0 ± 1^d^	89.4 ± 1^d^	91.1 ± 1^d^	97.3 ± 1^c^	105 ± 2^b^	108 ± 1^b^	113 ± 2^a^	90.5 ± 2^b^	92.8 ± 1^b^	95.3 ± 2^b^	110 ± 4^a^	107 ± 3^a^	0.011	<0.001	<0.001
Homo-cysteine	53.2 ± 3^a^	37.4 ± 3^b^	43.8 ± 3^ab^	45.0 ± 4^ab^	33.9 ± 2^b^	47.8 ± 2^a^	48.8 ± 2^a^	46.5 ± 2^a^	43.3 ± 2^a^	47.4 ± 2^a^	46.1 ± 2^a^	41.6 ± 3	40.9 ± 3	46.4 ± 3	49.2 ± 4	45.9 ± 4	0.015	<0.001	0.406
Ile	62.2 ± 2^ab^	63.9 ± 2^ab^	68.6 ± 2^a^	60.8 ± 2^b^	48.5 ± 1^f^	47.9 ± 1^f^	54.6 ± 1^e^	61.1 ± 1^d^	71.6 ± 2^c^	77.9 ± 1^b^	85.7 ± 2^a^	59.0 ± 1^b^	61.3 ± 1^b^	58.5 ± 1^b^	71.5 ± 2^a^	69.1 ± 2^a^	0.027	<0.001	<0.001
Leu^1^	—	—	—	—	—	—	—	—	—	—	—	119 ± 3^b^	120 ± 3^b^	117 ± 2^b^	134 ± 4^a^	130 ± 3^a^	—	—	0.001
Lys	130 ± 8^b^	134 ± 7^b^	166 ± 7^a^	140 ± 7^ab^	108 ± 4^e^	102 ± 4^e^	119 ± 4^d^	143 ± 4^c^	165 ± 5^b^	175 ± 5^ab^	184 ± 6^a^	137 ± 5	139 ± 4	136 ± 5	150 ± 7	151 ± 5	0.003	<0.001	0.079
Met	59.9 ± 2^a^	54.2 ± 2^ab^	53.1 ± 2^ab^	50.1 ± 2^b^	43.7 ± 1^e^	40.5 ± 1^f^	49.2 ± 1^d^	55.5 ± 1^c^	62.0 ± 1^b^	63.5 ± 1^ab^	65.0 ± 2^a^	52.9 ± 2^ab^	53.9 ± 2^ab^	50.8 ± 1^b^	56.6 ± 2^ab^	57.4 ± 2^a^	0.045	<0.001	0.021
Phe	64.7 ± 2^b^	67.1 ± 2^ab^	72.3 ± 2^a^	67.4 ± 2^ab^	64.5 ± 1^bc^	60.4 ± 1^d^	62.6 ± 1^cd^	66.3 ± 1^b^	72.0 ± 1^a^	73.9 ± 1^a^	75.4 ± 1^a^	63.0 ± 2^c^	66.2 ± 1^bc^	66.5 ± 1^abc^	73.5 ± 2^a^	70.2 ± 2^ab^	0.020	<0.001	0.002
Pro^1^	—	—	—	—	—	—	—	—	—	—	—	163 ± 4^b^	168 ± 4^b^	164 ± 4^b^	195 ± 8^a^	189 ± 7^a^	—	—	0.005
Ser	139 ± 8	139 ± 7	155 ± 8	160 ± 9	146 ± 5^bc^	131 ± 4^d^	138 ± 5^cd^	148 ± 5^abc^	159 ± 5^a^	159 ± 5^a^	157 ± 5^ab^	123 ± 3^b^	128 ± 4^b^	124 ± 4^b^	187 ± 18^a^	194 ± 15^a^	0.143	<0.001	<0.001
Tau	102 ± 4^b^	110 ± 4^ab^	122 ± 4^a^	119 ± 4^a^	112 ± 3^bc^	101 ± 3^d^	104 ± 3^cd^	116 ± 3^b^	125 ± 3^a^	121 ± 3^ab^	115 ± 3^bc^	105 ± 3^b^	99 ± 3^b^	101 ± 2^b^	128 ± 5^a^	135 ± 5^a^	0.004	<0.001	<0.001
Thr	145 ± 7	136 ± 6	137 ± 6	136 ± 6	129 ± 4^bc^	116 ± 4^d^	124 ± 4^cd^	137 ± 4^b^	151 ± 4^a^	155 ± 4^a^	158 ± 4^a^	126 ± 4^c^	131 ± 4^bc^	126 ± 4^c^	159 ± 9^ab^	154 ± 7^a^	0.699	<0.001	0.005
Trp	68.1 ± 3^b^	66.1 ± 2^b^	86.5 ± 3^a^	69.0 ± 3^b^	68.5 ± 2^c^	69.2 ± 2^bc^	72.0 ± 2^abc^	73.3 ± 2^ab^	74.9 ± 2^a^	74.5 ± 2^a^	74.6 ± 2^a^	69.0 ± 2	71.0 ± 2	71.1 ± 2	77.0 ± 3	73.8 ± 2	<0.001	<0.001	0.184
Tyr	54.5 ± 2	55.7 ± 2	58.5 ± 2	53.5 ± 2	54.1 ± 1^b^	48.4 ± 1^c^	50.6 ± 1^bc^	53.6 ± 1^b^	59.3 ± 1^a^	60.7 ± 1^a^	62.1 ± 1^a^	50.1 ± 1^b^	49.4 ± 1^b^	49.4 ± 1^b^	65.5 ± 4^a^	63.2 ± 3^a^	0.455	<0.001	<0.001
Val	165 ± 5	172 ± 5	181 ± 5	164 ± 5	141 ± 3^ef^	135 ± 3^f^	147 ± 3^e^	163 ± 3^d^	186 ± 4^c^	201 ± 3^b^	221 ± 4^a^	163 ± 4^bc^	167 ± 3^bc^	162 ± 3^c^	183 ± 6^a^	177 ± 4^ab^	0.048	<0.001	0.002
IDAAs	1042 ± 31	1040 ± 29	1137 ± 29	1048 ± 31	905 ± 17^d^	861 ± 17^e^	945 ± 19^d^	1051 ± 18^c^	1175 ± 22^b^	1231 ± 19^b^	1296 ± 22^a^	997 ± 23^b^	1022 ± 18^b^	1003 ± 19^b^	1164 ± 38^a^	1146 ± 28^a^	0.056	<0.001	<0.001
DAAs	2193 ± 48^a^	1986 ± 46^b^	2098 ± 45^ab^	2136 ± 50^ab^	2005 ± 33^c^	1851 ± 35^d^	1955 ± 35^c^	2145 ± 35^b^	2280 ± 35^a^	2272 ± 35^a^	2215 ± 35^ab^	1965 ± 29^b^	1982 ± 23^b^	1961 ± 28^b^	2349 ± 73^a^	2260 ± 61^a^	0.018	<0.001	<0.001

Abbreviations: AA, amino acid; DAA, dispensable amino acid; IDAA, indispensable amino acid; Pulse15, Pulse30, Pulse45, grain-free diets with increasing pulse inclusion.

^a,b,c^Values that do not share a common letter within rows are significantly different at *P* < 0.05.

1 AAs with significant treatment × time interaction effects are not displayed.

**TABLE 3:** Whole blood AA concentrations (lsmeans ± SEM nmol/mL) across the main effects of treatment (control, Pulse15, Pulse30, and Pulse45), time (0-fasted to 180 min), and week (2–20).

AA	Treatment				Time (min)							Week					*P*		
	Control	Pulse15	Pulse30	Pulse45	0	15	30	60	90	120	180	2	4	8	16	20	Treatment	Time	Week
*n*	197	209	224	164	127	110	112	113	114	110	108	170	166	160	153	145			
Ala	419 ± 14^a^	344 ± 13^b^	378 ± 13^ab^	366 ± 13^b^	351 ± 7^c^	329 ± 8^d^	348 ± 7^c^	389 ± 7^b^	409 ± 7^a^	415 ± 8^a^	399 ± 8^ab^	396 ± 12	393 ± 11	274 ± 13	359 ± 9	363 ± 9	0.001	<0.001	0.033^1^
Arg	154 ± 4^b^	182 ± 4^a^	188 ± 4^a^	193 ± 4^a^	173 ± 2^c^	166 ± 2^d^	173 ± 2^c^	181 ± 2^b^	186 ± 2^ab^	188 ± 2^a^	187 ± 2^ab^	177 ± 3	174 ± 4	178 ± 4	180 ± 3	187 ± 3	<0.001	<0.001	0.130
Asn	26.9 ± 1^a^	22.5 ± 1^b^	24.8 ± 1^ab^	26.6 ± 1^a^	19.6 ± 0.4^e^	19.7 ± 0.4^e^	21.7 ± 0.5^d^	25.3 ± 0.5^c^	28.3 ± 0.6^b^	30.2 ± 0.9^ab^	31.8 ± 0.8^a^	24.7 ± 0.7	25.1 ± 0.7	24.5 ± 0.8	25.7 ± 0.6	26.0 ± 0.7	0.005	<0.001	0.400
Asp	53.9 ± 2	54.6 ± 2	56.5 ± 2	51.4 ± 2	51.1 ± 1^d^	50.2 ± 1^d^	52.7 ± 1^cd^	54.2 ± 1^bc^	55.3 ± 1^abc^	57.1 ± 1^ab^	58.1 ± 1^a^	57.0 ± 2	53.3 ± 2	52.7 ± 2	52.4 ± 1	55.0 ± 1	0.261	<0.001	0.141
Cystine	3.67 ± 0.2^ab^	3.92 ± 0.1^a^	3.34 ± 0.1^bc^	2.93 ± 0.2^c^	3.56 ± 0.1^a^	3.66 ± 0.1^a^	3.60 ± 0.1^a^	3.55 ± 0.1^ab^	3.34 ± 0.1^bc^	3.32 ± 0.1^c^	3.25 ± 0.1^c^	3.52 ± 0.1	3.57 ± 0.2	3.28 ± 0.1	3.39 ± 0.1	3.58 ± 0.1	<0.001	<0.001	0.473
Gln	624 ± 14^a^	547 ± 13^b^	558 ± 13^b^	621 ± 14^a^	577 ± 9	578 ± 9	585 ± 9	599 ± 9	597 ± 9	593 ± 9	583 ± 9	593 ± 11	586 ± 12	583 ± 12	594 ± 10	580 ± 10	<0.001	0.029^1^	0.765
Glu	165 ± 8^b^	197 ± 7^a^	182 ± 7^ab^	168 ± 7^b^	176 ± 4^bc^	173 ± 4^c^	175 ± 4^bc^	177 ± 4^bc^	178 ± 4^abc^	181 ± 4^ab^	186 ± 4^a^	182 ± 8	177 ± 7	185 ± 9	172 ± 6	174 ± 6	0.009	0.004	0.744
Gly	255 ± 7	255 ± 7	251 ± 7	261 ± 7	235 ± 4^c^	215 ± 4^e^	226 ± 4^d^	261 ± 5^b^	280 ± 5^a^	287 ± 5^a^	284 ± 4^a^	249 ± 6	255 ± 7	257 ± 7	254 ± 5	262 ± 6	0.836	<0.001	0.516
His	99.0 ± 1^ab^	94.1 ± 1^b^	102 ± 1^a^	97.3 ± 2^ab^	92.7 ± 0.9^e^	91.8 ± 1^e^	93.0 ± 0.8^e^	95.7 ± 1^d^	100 ± 1^c^	104 ± 1^b^	109 ± 1^a^	98.9 ± 1	98.7 ± 1	97.8 ± 2	96.5 ± 1	98.5 ± 1	0.002	<0.001	0.602
Ile	58.6 ± 1^ab^	60.3 ± 1^ab^	62.8 ± 1^a^	58.0 ± 1^b^	49.7 ± 0.8^f^	48.7 ± 0.9^f^	52.6 ± 0.8^e^	57.1 ± 0.8^d^	64.2 ± 1^c^	70.3 ± 1^b^	76.9 ± 1^a^	60.4 ± 1	60.1 ± 1	58.5 ± 1	59.5 ± 1	61.1 ± 1	0.026	<0.001	0.287
Leu	131 ± 3^a^	115 ± 3^b^	121 ± 3^b^	113 ± 3^b^	97.3 ± 2^f^	96.2 ± 2^f^	105 ± 2^e^	114 ± 2^d^	129 ± 2^c^	142 ± 2^b^	155 ± 3^a^	123 ± 2	118 ± 2	117 ± 2	118 ± 2	122 ± 2	<0.001	<0.001	0.095
Lys^2^	—	—	—	—	—	—	—	—	—	—	—	226 ± 5	225 ± 5	225 ± 5	215 ± 5	221 ± 5	—	—	0.397
Met^2^	—	—	—	—	—	—	—	—	—	—	—	46.6 ± 1^ab^	46.1 ± 1^ab^	43.7 ± 1^b^	44.9 ± 1^ab^	48.0 ± 1^a^	—	—	0.008
Phe	63.9 ± 1^b^	66.1 ± 1^ab^	69.4 ± 1^a^	65.8 ± 1^ab^	63.0 ± 1^cd^	61.1 ± 1^d^	62.5 ± 1^d^	65.6 ± 1^c^	68.5 ± 1^b^	71.2 ± 1^ab^	72.1 ± 1^a^	68.9 ± 1^a^	66.6 ± 1^ab^	66.0 ± 1^ab^	63.8 ± 1^b^	66.2 ± 1^ab^	0.011	<0.001	0.006
Pro	—	—	—	—	—	—	—	—	—	—	—	156 ± 3	157 ± 3	152 ± 4	155 ± 3	158 ± 3	—	—	0.709
Ser	134 ± 4^b^	133 ± 3^b^	141 ± 3^ab^	149 ± 4^a^	138 ± 2^bc^	130 ± 2^d^	133 ± 2^cd^	139 ± 2^b^	143 ± 2^ab^	146 ± 2^a^	147 ± 2^a^	142 ± 3	141 ± 3	140 ± 4	134 ± 3	140 ± 3	0.006	<0.001	0.192
Tau^3^	188 ± 7	198 ± 7	196 ± 7	185 ± 8	177 ± 4^c^	184 ± 4^bc^	190 ± 4^ab^	197 ± 4^a^	198 ± 4^a^	198 ± 4^a^	196 ± 4^a^	192 ± 8^ab^	175 ± 7^b^	186 ± 7^ab^	203 ± 7^a^	202 ± 6^a^	0.561	<0.001	0.018
Thr	143 ± 4	135 ± 4	137 ± 4	136 ± 4	134 ± 2^cd^	125 ± 2^e^	129 ± 2^de^	136 ± 2^c^	142 ± 2^b^	148 ± 2^ab^	152 ± 2^a^	139 ± 3	138 ± 4	135 ± 4	137 ± 3	141 ± 3	0.540	<0.001	0.598
Trp	36.9 ± 1^b^	35.0 ± 1^b^	42.8 ± 1^a^	35.8 ± 1^b^	35.0 ± 1^d^	36.0 ± 1^cd^	37.1 ± 1^bc^	38.5 ± 1^ab^	38.7 ± 1^ab^	39.1 ± 1^ab^	38.9 ± 1^a^	38.0 ± 1	38.4 ± 1	37.0 ± 1	37.1 ± 1	37.7 ± 1	<0.001	<0.001	0.765
Tyr	61.4 ± 1^a^	58.8 ± 1^ab^	59.9 ± 1^ab^	56.3 ± 1^b^	58.7 ± 1^bc^	55.8 ± 1^d^	57.1 ± 1^cd^	58.6 ± 1^bc^	60.0 ± 1^ab^	61.6 ± 1^a^	62.1 ± 1^a^	61.5 ± 1^a^	60.1 ± 1^ab^	56.7 ± 1^b^	57.0 ± 1^b^	60.3 ± 1^ab^	0.050	<0.001	0.001
Val	151 ± 3^b^	156 ± 3^ab^	164 ± 3^a^	152 ± 2^ab^	136 ± 2^ef^	132 ± 2^f^	139 ± 2^e^	149 ± 2^d^	163 ± 2^c^	176 ± 2^b^	194 ± 3^a^	163 ± 3^a^	157 ± 3^ab^	154 ± 3^ab^	150 ± 2^b^	154 ± 2^ab^	0.031	<0.001	0.013
IDAA	1092 ± 20^b^	1101 ± 19^b^	1177 ± 18^a^	1113 ± 20^ab^	1026 ± 13^ef^	991 ± 14^f^	1041 ± 13^e^	1103 ± 13^d^	1173 ± 13^c^	1229 ± 14^b^	1283 ± 14^a^	1133 ± 17	1121 ± 17	1112 ± 18	1103 ± 16	1136 ± 15	0.008	<0.001	0.398
DAA	2104 ± 29^a^	1951 ± 28^b^	2001 ± 28^ab^	2043 ± 30^ab^	1914 ± 19^cd^	1848 ± 21^d^	1919 ± 20^c^	2053 ± 20^b^	2128 ± 20^a^	2160 ± 20^a^	2151 ± 21^a^	2054 ± 26	2011 ± 28	2014 ± 30	2018 ± 24	2027 ± 22	0.003	<0.001	0.742

Abbreviations: AA, amino acid; DAA, dispensable amino acid; IDAA, indispensable amino acid; Pulse15, Pulse30, Pulse45, grain-free diets with increasing pulse inclusion.

^a,b,c^Values that do not share a common letter within rows are significantly different at *P* < 0.05.

1 Although the null hypothesis was rejected in the F-test (*P* = 0.033), no significant differences were observed between weeks for whole blood Ala when pairwise comparisons were analyzed using the Tukey-Kramer adjustment.

2 AAs with significant treatment × time interaction effects are not displayed.

3 Week-1 was included as a covariate for Tau analysis.

All plasma (Table 2) and whole blood (Table 3) AAs that demonstrated a significant treatment effect, responded similarly: that is, alanine was greater in the control diet than that in Pulse15 and Pulse45; Arg was lowest in the control diet; Gln was greater in the control diet (and Pulse45 in whole blood) than that in Pulse15 and Pulse30; His was greater in Pulse30 than that in Pulse15; Ile was greater in Pulse30 than that in Pulse45; Phe was greater in Pulse30 than that in the control diet; and Trp was the greatest in Pulse30 in both plasma and whole blood (*P* < 0.05). Additionally, in plasma, GSH was the lowest in the control diet, homocysteine was greater in the control diet than that in Pulse15, Lys was greater in Pulse30 than that in Pulse15 and the control diet, Met was greater in the control diet than that in Pulse45, and Tau was greater in Pulse30 and Pulse45 than that in the control diet (*P* < 0.05). In whole blood, Asn was greater in the control diet and Pulse45 than that in Pulse15, cystine was greater in Pulse15 than that in Pulse30 and Pulse45, glutamic acid was greater in Pulse15 than that in the control diet and Pulse45, Leu was the greatest in the control diet, Ser was greater in Pulse45 than that in the control diet and Pulse15, tyrosine was greater in the control diet than that in Pulse45, and valine was greater in Pulse30 than that in the control diet (*P* < 0.05). Total dispensable amino acids (DAAs) were greater in the control diet than those in Pulse15 in plasma and whole blood, and total indispensable amino acids (IDAAs) were greater in Pulse30 than those in Pulse15 and the control diet in whole blood (*P* < 0.05).

Most AAs were influenced by the number of weeks the dogs had been fed the treatments, and all were affected by the time blood was sampled postmeal. Plasma Arg, Gly, His, Ile, Phe, Ser, Tau, Thr, Tyr, Val, and total IDAAs and DAAs increased over both time postmeal and week (*P* < 0.05), and Asn, Leu, and Pro also increased over week. Orthogonal contrasts revealed plasma Met, Tau, Arg, Ser, and Leu had linear trends (*P* < 0.05) where Met and Leu decreased as pulse inclusion increased and Tau, Arg, and Ser increased as pulse inclusion increased. In addition, plasma Asn, Trp, Ala, Gln, Ile, Lys, Phe, Pro, Val, homocysteine, GSH, and total DAA had quadratic trends as pulse inclusion increased (*P* < 0.05). All whole blood AAs except Gln had a time postmeal effect, and only Met, Phe, Tau, Tyr, and Val had a week effect. Of the AAs with a time postmeal effect, including total IDAA and DAA, all of them increased over time postmeal, with the exception of cystine, which decreased over time postmeal (*P* < 0.05). Finally, orthogonal contrasts revealed whole blood Met, Leu, and Tyr decreased linearly as pulse inclusion increased (*P* < 0.05) and Ser increased linearly as pulse inclusion increased (*P* < 0.05). In addition, whole blood cystine, Trp, Glu, Arg, Ala, Ile, Asn, Gln, Lys, Phe, Pro, Val, and total DAA had quadratic trends (*P* < 0.05).

Correlations between nutritional analysis of AAs and foodomic analysis of AAs ranged from 0.02 for Ile to 0.8 for Phe (Supplemental Table 1). Both methods followed similar trends in terms of their correlations with plasma and whole blood AAs. In general, the correlations were low for plasma and whole blood Val (0.01–0.08) and Ile (0.05–0.25) and high for plasma and whole blood Arg (0.72–0.97) and Met (0.62–0.97) (Supplemental Table 1).

### Foodomics

All food compounds with a VIP > 1.00 associated with plasma AA, whole blood AA, or SAAs at all time points are presented in Table 4. Of the top compounds in plasma for all time points, 29 were phospholipids, 14 were carnitines, 11 were AA and/or their derivatives, 5 were polyphenols, 2 were short-chain fatty acids, 2 were sphingomyelins, 1 was an oligosaccharide, and 1 was a vitamin. Of the top compounds in whole blood for all time points, 13 were phospholipids, 11 were AAs and their derivatives, 9 were carnitines, 4 were polyphenols, 3 were sphingomyelins, 1 was a short-chain fatty acid, and 1 was a vitamin. Of the top compounds in plasma for SAA at all time points, 18 were phospholipids, 17 were AAs and their derivatives, 11 were carnitines, 8 were polyphenols, 3 were oligosaccharides, 3 were vitamins, and 2 were sphingomyelins.

**TABLE 4:** Top compounds with a VIP > 1.00 associated with predicting either all plasma AAs over time, all whole blood AAs over time, or the SAAs over time.

	All plasma AAs	All whole blood AAs	Plasma SAAs
1	PC38:0AA	Propionic acid	Carnosine
2	LYSOC26:0	Pyruvic acid	PC30:0AE
3	PC36:1AE	PC42:0AE	Indole acetic acid
4	C6	Leucine	Vitamin B-2
5	PC42:0AA	PC40:3AE	PC40:2AA
6	C18	Citrulline	Stachyose
7	C5MDC	C16	C16:2OH
8	PC36:0AE	C12DC	C5
9	PC42:1AE	Spermine	Ferulic acid
10	C18:2	C18:2	Proline
11	C6:1	Vitamin B-3	Gallic acid
12	PC38:1AE	PC40:1AA	Methionine sulfoxide
13	PC30:2AE	C3:1	Glucose
14	PC40:1AE	C5MDC	Creatinine
15	Citrulline	Sarcosine	Caffeic acid
16	PC42:0AE	PC32:2AA	LYSOC26:1
17	LYSOC24:0	C16:2	34-Dihydroxybenzoic acid
18	PC40:3AE	Gallocatchin	PC38:5AE
19	Vanillic acid	LYSOC28:0	Creatine
20	Raffinose	Methylmalonic acid	Glyceric acid
21	PC40:2AE	C18	LYSOC28:0
22	PC42:2AE	Creatine	Methylmalonic acid
23	PC40:4AE	22:3SM	PC40:4AA
24	Pyruvic acid	PC28:1AA	C16:2
25	C3	Vanillic acid	26:1SM
26	Cis-hydroxyproline	PC36:5AA	Verbascose
27	C8	PC36:4AA	C3OH
28	PC30:1AE	26:0SM	PC44:6AE
29	C2	Glyceric acid	Vanillin
30	C5:1	PC30:2AA	C4:1
31	PC42:2AA	PC36:3AA	Methionine
32	Protocatechuic aldehyde	Protocatechuic aldehyde	Sucrose
33	PC38:1AA	PC40:2AE	PC38:6AA
34	Propionic acid	PC30:0AA	Phenylalanine
35	Glutamic acid	C3OH	PC40:5AA
36	Vitamin B-7	22:1SMOH	p-Coumaric acid
37	PC38:0AE	3,4-Dihydroxybenzoic acid	Lactic acid
38	PC42:5AA	Methionine	Betaine
39	Serotonin	C14:2	PC32:2AA
40	PC42:6AA	Creatinine	Tyramine
41	C16	PC38:0AA	PC38:5AA
42	C14:2	Proline	PC36:5AA
43	PC44:4AE	—	Choline
44	PC34:0AE	—	PC36:2AA
45	Arginine	—	C4
46	Aspartic acid	—	PC36:3AA
47	C12:1	—	Gallocatchin
48	PC32:0AA	—	PC42:6AA
49	PC36:0AA	—	Carnitine
50	Methylhistidine	—	C10:1
51	Succinic acid	—	PC36:4AA
52	Butyric acid	—	Oxalic acid
53	PC32:1AA	—	Vitamin B-5
54	Shikimic acid	—	PC40:6AE
55	Gallocatchin	—	22:3SM
56	C5DC	—	C12DC
57	p-Coumaric acid	—	Valeric acid
58	Sucrose	—	Vitamin B-3
59	C14:2OH	—	PC36:4AE
60	16:1SM	—	Protocatechuic aldehyde
61	Vanillin	—	PC38:4AA
62	22:1SMOH	—	PC36:6AA
63	PC44:6AE	—	C7DC
64	LYSOC20:3	—	—
65	PC38:2AE	—	—

Abbreviations: AA, amino acid; SAA, sulfur amino acid; VIP, variable importance of projection.

MetaboAnalyst 5.0 was not able to recognize 8 compounds in Table 4. The MSEA revealed that the most enriched pathway in the list of compounds associated with plasma AAs across all time points was the urea cycle (*P* = 0.008). The most enriched pathway in the list of compounds associated with whole blood AAs across all time points was Gly and Ser metabolism (*P* = 0.047). In addition, the most enriched pathway in the list of compounds associated with plasma SAA across all time points was betaine metabolism; however, this was not significant (*P* = 0.705).

## Discussion

The objective of this study was to evaluate the postprandial plasma and whole blood AA response in dogs fed a control, corn-inclusive diet and 3 grain-free diets with increasing whole pulse inclusion, ≤45%, and to correlate that with compounds found in the diets. In partial support of our hypothesis, plasma Leu was greater in dogs fed control diet than that in dogs fed Pulse15 and Pulse45 at certain time points, but not greater than Pulse30 at any timepoint, despite Pulse30 having the lowest Leu concentrations in the diet. The control diet contained corn gluten meal, a concentrated source of protein, which contains ∼6 times the amount of Leu than whole yellow peas, green lentils, and chickpeas [13]. Furthermore, corn gluten meal contains >7 times the amount of Met than pulses that aligns with findings in whole blood where, again, dogs fed control diet had greater Met concentrations than dogs fed Pulse15 and Pulse45 at certain time points. Despite dogs fed Pulse30 having similar concentrations of Leu and Met at all time points than dogs fed the control diet, the overall trend for these AA was still linear, according to orthogonal contrasts. The Met and Leu content was the greatest in the control diet, and the standardized AA digestibility scores for Met and Leu are both >96% DMB for corn gluten meal [13]. Owing to the inherent nature of pulse ingredients, the total dietary fiber increased with pulse inclusion. However, soluble fiber was highest in Pulse30. In humans, soluble fiber is known to delay the time to peak glucose in the blood, likely owing to increased viscosity and delayed gastric emptying [27]. In dogs, pulse ingredients have also been shown to delay time to peak glucose and insulin [28]. In a study done in healthy dogs consuming a low-protein diet (17% crude protein) with either supplementary insoluble fiber (cellulose) or soluble fiber (guar gum and sugar beet pulp), the combined 3-h AUC for plasma leucine and isoleucine was greater in dogs fed the soluble fiber [29]. The authors hypothesized that the increased production of fermentation-derived acetic and propionic acid was able to spare AAs. In unpublished data from this study, dogs fed Pulse30 had, numerically, the greatest concentration of propionic acid in the feces that was significantly greater than dogs fed the control diet, but not different from dogs fed Pulse15 or Pulse45, who were also not different from dogs fed the control diet. Although there are certainly many attributes of fiber that can influence AA digestion and absorption, it is possible that fermentation end products may be playing a role in this study given that AA concentrations in the pulse diets were similar but fiber components differed.

Ileal digestibility of AA is the greatest source of variability in AA bioavailability [30]. Reilly et al. [10] analyzed several pulse ingredients, including yellow peas, green lentils, and chickpeas, using the cecectomized rooster assay, and reported the highest AA concentrations for Arg, Leu, and Lys, each of which had a standardized AA digestibility of >84% DMB. On the contrary, both yellow peas and green lentils were considered deficient in Met given that they fell below the minimal requirement (0.26% DMB) of Met recommended for adult dogs at maintenance [3]. Furthermore, their standardized AA digestibility scores were below 80% DMB. This highlights why single ingredients are not fed alone, but as part of a food matrix in order to meet and exceed requirements. This, in part, aligns with findings in this study. We hypothesized that plasma and whole blood Lys would be greater in dogs fed all pulse diets than those fed the control diet but only reported that whole blood Lys was greater in dogs fed Pulse30 than that in dogs fed the control diet and Pulse15 at certain time points. This may suggest that Lys bioavailability differed between the pulse diets, despite having very similar amounts of total Lys in each of the pulse diets. This would be expected given that Lys is known to form Maillard products during extrusion, resulting in part of the total Lys measured being unavailable for use by the animal [31]. In unpublished data from cecectomized rooster digestibility analysis of these diets, despite having ∼1.4 times the reactive Lys in the pulse diets than that in the control diet, the digestibility of Lys did not differ among all 4 diets. Furthermore, the digestibility of every AA was not different among the 3 pulse diets, suggesting that differences reported in appearance of these AAs in the blood are not accounted for by differences in digestibility. These findings, along with other nonlinear results from this study, in terms of increasing AAs in the diets and expected increasing AAs in the blood, further highlights our initial argument that AA metabolism is dynamic and understanding digestibility, plasma appearance, or AA content in the diet alone does not provide a full picture. Furthermore, future studies should evaluate interactions between fiber components and AAs during processing and digestion and absorption in companion animals.

Different AAs play various important roles in metabolism. It has been well established that Leu, together with insulin, is necessary to stimulate protein synthesis in skeletal muscle [32]. Met’s major role in the body is as a methyl donor via S-adenosylmethionine (SAM) and as a precursor to both Cys and Tau [33]. Finally, Lys may be the first limiting AA in some canine diets and is involved in the synthesis of carnitine, using 4 methyl groups from SAM [34]. In addition to the interaction effects observed in these 3 IDAAs, when comparing the control diet with Pulse45, the most similar diets, with the exception of corn and whole pulse ingredients inclusion, plasma Met and whole blood Leu were greater in dogs fed the control diet and plasma and whole blood Arg were greater in dogs fed Pulse45. Despite these differences, when total IDAAs were compared, there were no differences in concentrations between dogs fed the control diet and dogs fed Pulse45, suggesting that there were unlikely to be differences in protein synthesis. In support of this, there were no differences in body composition between treatments [14]. Furthermore, all diets exceeded AAFCO recommendations for IDAA for adult dogs at maintenance; therefore, the changes that were observed in blood AA concentrations are due to the differences in AA profiles of the ingredients used and not an AA or protein deficiency. In addition, no dogs had any plasma or whole blood AA concentration that would be considered deficient.

Interestingly, although individual AA treatment differences were similar in plasma and whole blood, concentrations of whole blood AAs were more resistant to change over the 20 wk. Only 5 whole blood AAs were significantly different between weeks, and even then, none were different between week 2 and week 20, suggesting that whole blood may be a better long-term index. In contrast, the majority of plasma AAs increased over the 20 wk. Investigations into differences in plasma compared with whole blood are often focused on Tau, given that Tau deficiency can lead to DCM in dogs and cats [35,36]; however, other whole blood AAs are rarely reported in dogs. It has previously been established that whole blood Tau is more stable after a meal and during fasting in dogs compared with plasma [37]. In agreement with this, in this study, we report differences in plasma Tau but not whole blood Tau among treatments. More recent studies comparing Tau concentrations in plasma, whole blood, and muscle, report that concentrations depend on a variety of factors including size of the dog [38,39], Met+Cys concentrations in the diet [38], and specific tissue sampled [39]. Furthermore, McCauley et al. [39] report no strong correlations among plasma, whole blood, skeletal, and endomyocardial Tau concentrations. Although invasive, muscle biopsy may be better suited to more accurately assess a clinical Tau deficiency, given that taurine is known to be more concentrated in myocardial muscle. However, this is unlikely to occur, given ethical constraints, especially in a research setting, thus plasma and whole blood Tau may remain as our best attempt at measuring whole body Tau concentrations. Like Tau, GSH is a downstream metabolite of Met with oxidative capacity [40] and was also greater in dogs fed Pulse45. Given that there is a lot of regulation around the conversion of Cys to GSH, Tau, or pyruvate/inorganic sulfate [41], the dogs used in this study were healthy and not under oxidative stress, and all diets were sufficient in SAA, and the difference in GSH concentrations may not be biologically relevant. In addition, this is a group of conditioned, short-distance racing dogs, and moderate exercise can lead to improved ability to respond to oxidative stress [42].

We also report many differences in the food compounds that are predictive of plasma, whole blood, and SAA concentrations in the blood of the dogs. However, it is difficult to make conclusions given that there are some limitations when it comes to interpreting these type of data. First, the PLS regression is simply a list of compounds in the food that are predictive of all of the AAs measured in the blood across all diets, weeks, and dogs. Therefore, overall trends can be observed but individual compounds should not be called out as having an effect on plasma or whole blood AA concentrations. As such, we also performed the MSEA in order to group some of the compounds together. However, the available databases in MetaboAnalyst 5.0 only contain AA metabolic pathways; therefore, it is only capturing a subsample of the list of compounds. Finally, the laboratory analysis used for the foodomics profile is not the same as the Association of Official Analytical Communities (AOAC) protocols that are standard for pet food analysis [22], which explains the low correlations. The foodomics profile uses a nondestructive method that does not involve acid hydrolysis to release AAs from protein whereas the AOAC methods do. As such, there were unavoidable discrepancies in dietary AAs when analyzed using the 2 different methods, and the purpose of the foodomics analysis was not to quantify or replace AOAC methods. Despite these limitations, a number of interesting patterns emerged. Although the majority of compounds represented in each outcome were phospholipids (discussed further), these tended to appear much lower on the list when only the plasma SAA were included. Importantly, many cofactors and methylated compounds, such as betaine, creatine, carnitine, and choline, appeared on the list, whereas Met was the only AA directly involved in SAA metabolism that appeared on the list. Creatine and carnitine are the major methyl receivers from SAM [43], and choline via betaine is one of the routes for remethylation from homocysteine to methionine and has shown to be important during growth [44,45] and heat stress [46]. Furthermore, the importance of providing these methyl donors and receivers in grain-free dog diets was highlighted previously by our laboratory [24].

We also observed that in each group, the majority of compounds generated in the list belonged to the phospholipid family including phosphatidylcholines and lysophosphatidylcholines. Phosphotidylcholines are the most abundant phospholipids and are involved in regulating lipid and energy metabolism [47]. Lysophosphotidylcholines are involved in cholesterol synthesis and fatty acid oxidation [48]. One reason for their higher prevalence in the PLS regression output may simply be that this class of compounds were the most represented (∼40%) in the total number of compounds analyzed. Nevertheless, this result is intriguing, and although it would be unreasonable to try to pinpoint individual phospholipids, these results may be hypothesis generating given that the role of phospholipids in grain-free diets has largely been unexplored. In the only other study to explore foodomic profiles in dog food, Smith et al. [21] identified only 5 compounds in the lipid class that were lower in grain-free diets than those in traditional grain-inclusive diets, with the majority of the compounds the authors discussed being AAs and their derivatives. However, the diets used were commercially available, did not control for macronutrient or micronutrient targets, and were not fed to dogs in order to determine appearance of such compounds in the animal. Differences in blood phospholipid profiles have been reported in dogs with chronic enteropathy [19], dogs with anxiety [49], and obese dogs [50], to name a few. Therefore, the interaction between dietary and blood phospholipids may become useful in identifying common disorders in dogs. In human nutrition research, metabolomics are being widely applied to investigate the effects of dietary intervention on blood, fecal, and urine metabolomic profiles to understand how nutrient consumption affects our overall health, including identifying new biomarkers [51]. This avenue of research may be useful to the pet food industry as new ingredients and dietary trends continue to emerge.

In conclusion, several plasma and whole blood AA concentrations differed between the 2 most similar diets (in terms of inclusion of every ingredient other than corn or pulses): the control diet and Pulse45. In general, plasma Met and Ala and whole blood Leu, free cysteine, and Tyr were greater in the control diet whereas plasma Arg, Tau, and GSH and whole blood Arg and Ser were greater in Pulse45. The differences were likely due to differences in the AA profiles of corn and corn gluten meal compared with peas, lentils, chickpeas, and beans, given that all diets were formulated to meet similar macronutrient and micronutrient targets and exceed AAFCO minimum recommendations, and the dogs were all healthy. Additionally, whole blood AAs were more resistant to change over weeks, and taken together with results from previous studies, this may have implications when identifying what type of sample should be collected when identifying changes in AA concentrations in clinical cases. Finally, compounds belonging to the phospholipid family were well represented in the PLS regression, suggesting that they play a role in predicting blood AA concentrations. Foodomic data such as these may help generate hypotheses and open the door to new research in the future of pet food as more and more novel ingredients continue to emerge.

## Author contributions

The authors’ responsibilities were as follows – SB, PS, AKS: designed the study; SB, PS: conducted the research; SB, DJS: analyzed all data; SB: wrote the paper; AKS, JSB: formulated the experimental diets and Champion Petfoods manufactured the diets; AKS: has primary responsibility for the final content; and all authors: read and approved the final manuscript.

## Conflicts of interest

SB and PS have no conflicts of interest. DJS is employed by Trouw Nutrition. JSB is employed by Champion Petfoods. AKS reports a relationship with Champion Petfoods that includes board membership, consulting or advisory, funding grants, speaking and lecture fees, and travel reimbursement; is a former employee of Procter & Gamble and Mars Petcare; is the Champion Petfoods Chair in Nutrition, Physiology and Metabolism; has received honoraria and research funding from various pet food manufacturers and ingredient suppliers in addition to provincial and federal granting agencies and serves on the Trouw Nutrition and Champion Petfoods Scientific Board.

## Funding

Champion Petfoods funded the study; however, this financial support did not influence the findings or conclusions of this study.

## References

[1] Boye J., Zare F., Pletch A. (2010). Pulse proteins: processing, characterization, functional properties and applications in food and feed. Food Res. Int..

[2] Tiwari B.K., Singh N. (2012).

[3] National Research Council (NRC) (2006).

[4] McCrory M.A., Hamaker B.R., Lovejoy J.C., Eichelsdoerfer P.E. (2010). Pulse consumption, satiety, and weight management. Adv. Nutr..

[5] Mansilla W.D., Marinangeli C.P.F., Ekenstedt K.J., Larsen J.A., Aldrich G., Columbus D.A. (2019). Special topic: the association between pulse ingredients and canine dilated cardiomyopathy: addressing the knowledge gaps before establishing causation. J. Anim. Sci..

[6] Hugman J., Wang L.F., Beltranena E., Htoo J.K., Zijlstra R.T. (2021). Nutrient digestibility of heat-processed field pea in weaned pigs. Anim. Feed Sci. Technol..

[7] Nosworthy M.G., Franczyk A.J., Medina G., Neufeld J., Appah P., Utioh A. (2017). Effect of processing on the *in vitro* and *in vivo* protein quality of yellow and green split peas (*Pisum sativum*). J. Agric. Food Chem..

[8] Bandegan A., Golian A., Kiarie E., Payne R.L., Crow G.H., Guenter W. (2011). Standardized ileal amino acid digestibility in wheat, barley, pea and flaxseed for broiler chickens. Can. J. Anim. Sci..

[9] Johnson M.L., Parsons C.M., Fahey G.C., Merchen N.R., Aldrich C.G. (1998). Effects of species raw material source, ash content, and processing temperature on amino acid digestibility of animal by-product meals by cecectomized roosters and ileally cannulated dogs. J. Anim. Sci..

[10] Reilly L.M., von Schaumburg P.C., Hoke J.M., Davenport G.M., Utterback P.L., Parsons C.M. (2020). Macronutrient composition, true metabolizable energy and amino acid digestibility, and indispensable amino acid scoring of pulse ingredients for use in canine and feline diets. J. Anim. Sci..

[11] Pezzali J.G., Acuff H.L., Henry W., Alexander C., Swanson K.S., Aldrich C.G. (2020). Effects of different carbohydrate sources on taurine status in healthy Beagle dogs. J. Anim. Sci..

[12] Donadelli R.A., Pezzali J.G., Oba P.M., Swanson K.S., Coon C., Varney J. (2020). A commercial grain-free diet does not decrease plasma amino acids and taurine status but increases bile acid excretion when fed to Labrador Retrievers. Transl. Anim. Sci..

[13] Reilly L.M., He F., Clark L., de Godoy M.R.C. (2021). Longitudinal assessment of taurine and amino acid concentrations in dogs fed a green lentil diet. J. Anim. Sci..

[14] Singh P., Banton S., Raheb S., Templeman J.R., Saunders-Blades J., Kostiuk D. (2023). The pulse of it: dietary inclusion of up to 45% whole pulse ingredients with chicken meal and pea starch in a complete and balanced diet does not affect cardiac function, fasted sulfur amino acid status, or other gross measures of health in adult dogs. J. Nutr..

[15] Clish C.B. (2015). Metabolomics: an emerging but powerful tool for precision medicine, Cold Spring Harb. Mol. Case Stud..

[16] Rankovic A., Godfrey H., Grant C.E., Shoveller A.K., Bakovic M., Kirby G. (2023). Serum metabolomic analysis of the dose-response effect of dietary choline in overweight male cats fed at maintenance energy requirements. PLoS One.

[17] Adin D.B., Haimovitz D., Freeman L.M., Rush J.E. (2022). Untargeted global metabolomic profiling of healthy dogs grouped on the basis of grain inclusivity of their diet and of dogs with subclinical cardiac abnormalities that underwent a diet change. Am. J. Vet. Res..

[18] Viant M.R., Ludwig C., Rhodes S., Günther U.L., Allaway D. (2007). Validation of a urine metabolome fingerprint in dog for phenotypic classification. Metabolomics.

[19] Walker H.K., Boag A.M., Ottka C., Lohi H., Handel I., Gow A.G., Mellanby R.J. (2022). Serum metabolomic profiles in dogs with chronic enteropathy. J. Vet. Intern. Med..

[20] Bordoni A., Capozzi F. (2015). The foodomics approach for discovering biomarkers of food consumption in nutrition studies. Curr. Opin. Food Sci..

[21] Smith C.E., Parnell L.D., Lai C.Q., Rush J.E., Freeman L.M. (2021). Investigation of diets associated with dilated cardiomyopathy in dogs using foodomics analysis. Sci. Rep..

[22] (2019). Association of American Feed Control Officials (AAFCO), AAFCO manual, AAFCO.

[23] Bidlingmeyer B.A., Cohen S.A., Tarvin T.L. (1984). Rapid analysis of amino acids using pre-column derivatization. J. Chromatogr..

[24] Banton S., Pezzali J.G., Verbrugghe A., Bakovic M., Wood K.M., Shoveller A.K. (2021). Addition of dietary methionine but not dietary taurine or methyl donors/receivers to a grain-free diet increases postprandial homocysteine concentrations in adult dogs. J. Anim. Sci..

[25] Vester B., Rasmussen K. (1991). High performance liquid chromatography method for rapid and accurate determination of homocysteine in plasma and serum. Eur. J. Clin. Chem. Clin. Biochem..

[26] Pfeiffer C.M., Huff D.L., Gunter E.W. (1999). Rapid and accurate HPLC assay for plasma total homocysteine and cysteine in a clinical laboratory setting. Clin. Chem..

[27] Jenkins D.J., Wolever T.M., Leeds A.R., Gassull M.A., Haisman P., Dilawari J. (1978). Dietary fibres, fibre analogues, and glucose tolerance: importance of viscosity. Br. Med. J..

[28] Carciofi A.C., Takakura F.S., De-Oliveira L.D., Teshima E., Jeremias J.T., Brunetto M.A. (2008). Effects of six carbohydrate sources on dog diet digestibility and post-prandial glucose and insulin response. J. Anim. Physiol. Anim. Nutr. (Berl)..

[29] Wambacq W., Rybachuk G., Jeusette I., Rochus K., Wuyts B., Fievez V. (2016). Fermentable soluble fibres spare amino acids in healthy dogs fed a low-protein diet. BMC Vet. Res..

[30] Food and Agriculture Organization (FAO) (March 2013). Dietary protein quality evaluation in human nutrition: report of an FAO expert consultation. Food and Agriculture Organization of the United Nations.

[31] Hurrell R.F., Carpenter K.J. (1974). Mechanisms of heat damage in proteins: 4. The reactive lysine content of heat-damaged material as measured in different ways. Br. J. Nutr..

[32] Garlick P.J. (2005). The role of leucine in the regulation of protein metabolism. J. Nutr..

[33] Courtney-Martin G., Pencharz P.B., Dardevet D. (2016). The molecular nutrition of amino acids and proteins.

[34] Tanphaichitr V., Horne D.W., Broquist H.P. (1971). Lysine, a precursor of carnitine in the rat. J. Biol. Chem..

[35] Kittleson M.D., Keene B., Pion P.D., Loyer C.G. (1997). Results of the multicenter spaniel trial (MUST): taurine- and carnitine-responsive dilated cardiomyopathy in American cocker spaniels with decreased plasma taurine concentration. J. Vet. Intern. Med..

[36] Pion P.D., Kittleson M.D., Rogers Q.R., Morris J.G. (1987). Myocardial failure in cats associated with low plasma taurine: a reversible cardiomyopathy. Science.

[37] Gray K., Alexander L.G., Staunton R., Colyer A., Watson A., Fascetti A.J. (2016). The effect of 48-hour fasting on taurine status in healthy adult dogs. J. Anim. Physiol. Anim. Nutr. (Berl)..

[38] Tôrres C.L., Biourge V.C., Backus R.C. (2022). Plasma and whole blood taurine concentrations in dogs may not be sensitive indicators of taurine deficiency when dietary sulfur amino acid content is reduced. Front Vet Sci.

[39] McCauley S.R., Clark S.D., Leach S.B., Quest B.W., Streeter R.M. (2024). Evaluation of taurine and carnitine concentrations in whole blood, plasma, skeletal muscle and cardiac muscle in dogs. J. Anim. Physiol. Anim. Nutr (Berl)..

[40] Shimada K., Jong C.J., Takahashi K., Schaffer S.W. (2015). Role of ROS production and turnover in the antioxidant activity of taurine. Adv. Exp. Med. Biol..

[41] Stipanuk M.H. (2004). Role of the liver in regulation of body cysteine and taurine levels: a brief review. Neurochem. Res..

[42] Huntingford J.L., Levine C.B., Mustacich D.J., Corrigan D., Downey R.L., Wakshlag J.J. (2014). The effects of low intensity endurance activity on various physiological parameters and exercise induced oxidative stress in dogs. J. Open, Vet. Med..

[43] Stead L.M., Brosnan J.T., Brosnan M.E., Vance D.E., Jacobs R.L. (2006). Is it time to reevaluate methyl balance in humans?. Am. J. Clin. Nutr..

[44] Robinson J.L., Bartlett R.K., Harding S.V., Randell E.W., Brunton J.A., Bertolo R.F. (2016). Dietary methyl donors affect in vivo methionine partitioning between transmethylation and protein synthesis in the neonatal piglet. Amino Acids.

[45] McBreairty L.E., Robinson J.L., Harding S.V., Randell E.W., Brunton J.A., Bertolo R.F. (2016). Betaine is as effective as folate at re-synthesizing methionine for protein synthesis during moderate methionine deficiency in piglets. Eur. J. Nutr..

[46] Liu W., Yuan Y., Sun C., Balasubramanian B., Zhao Z., An L. (2019). Effects of dietary betaine on growth performance, digestive function, carcass traits, and meat quality in indigenous yellow-feathered broilers under long-term heat stress. Animals (Basel).

[47] van der Veen J.N., Kennelly J.P., Wan S., Vance J.E., Vance D.E., Jacobs R.L. (2017). The critical role of phosphatidylcholine and phosphatidylethanolamine metabolism in health and disease. Biochim. Biophys. Acta Biomembr..

[48] Law S.H., Chan M.L., Marathe G.K., Parveen F., Chen C.H., Ke L.Y. (2019). An updated review of lysophosphatidylcholine metabolism in human diseases. Int. J. Mol. Sci..

[49] Puurunen J., Tiira K., Lehtonen M., Hanhineva K., Lohi H. (2016). Non-targeted metabolite profiling reveals changes in oxidative stress, tryptophan and lipid metabolisms in fearful dogs. Behav. Brain Funct..

[50] Söder J., Wernersson S., Dicksved J., Hagman R., Östman J.R., Moazzami A.A. (2019). Indication of metabolic inflexibility to food intake in spontaneously overweight Labrador Retriever dogs. BMC Vet. Res..

[51] Bertram H.C. (2023). NMR foodomics in the assessment of diet and effects beyond nutrients. Curr. Opin. Clin. Nutr. Metab. Care..

