# Supplementary material for: Postprandial Plasma and Whole Blood Amino Acids Are Largely Indicative of Dietary Amino Acids in Adult Dogs Consuming Diets with Increasing Whole Pulse Ingredient Inclusion
Source: J Nutr. 2024 Jul 16;154(9):2655–69. doi: 10.1016/j.tjnut.2024.07.023 (PMC11393166; doi:10.1016/j.tjnut.2024.07.023)
Supplement: multimedia component 1 [file mmc1.docx]

|  | Pearson Correlation Coefficient | | | | |
| --- | --- | --- | --- | --- | --- |
| Amino Acid | Nutritional AA vs Foodomic AA | Nutritional AA vs Plasma AA | Foodomic AA vs Plasma AA | Nutritional AA vs Whole Blood AA | Foodomic AA vs Whole Blood AA |
| Arginine | 0.51 | 0.95 | 0.74 | 0.97^*^ | 0.72 |
| Lysine | 0.09 | 0.46 | 0.37 | 0.66 | 0.26 |
| Methionine | 0.7 | 0.97^*^ | 0.62 | 0.92 | 0.69 |
| Phenylalanine | 0.8 | 0.65 | 0.07 | 0.72 | 0.18 |
| Taurine | 0.37 | 0.61 | 0.49 | 0.69 | 0.49 |
| Histidine | 0.35 | 0.85 | 0.58 | 0.93 | 0.64 |
| Isoleucine | 0.02 | 0.05 | 0.25 | 0.12 | 0.28 |
| Leucine | 0.3 | 0.78 | 0.26 | 0.81 | 0.25 |
| Threonine | 0.16 | 0.96^*^ | 0.21 | 0.97^*^ | 0.32 |
| Tryptophan | 0.41 | 0.4 | 0.47 | 0.24 | 0.39 |
| Tyrosine | 0.11 | 0.5 | 0.02 | 0.47 | 0.15 |
| Valine | 0.19 | 0.01 | 0.06 | 0.05 | 0.08 |

Supplemental Table 1. Correlations between the two different amino acid analyses (Foodomic AA and Nutritional AA) and their correlations with treatment lsmeans for plasma and whole blood AAs.

^*^ Indicates statistical significance at P<0.05
